# Gene prediction in metagenomic fragments: A large scale machine learning approach

**DOI:** 10.1186/1471-2105-9-217

**Published:** 2008-04-28

**Authors:** Katharina J Hoff, Maike Tech, Thomas Lingner, Rolf Daniel, Burkhard Morgenstern, Peter Meinicke

**Affiliations:** 1 Abteilung Bioinformatik, Georg-August-Universität Göttingen, Goldschmidtstr. 1, 37077 Göttingen, Germany; 2 Abteilung Genomische und Angewandte Mikrobiologie, Georg-August-Universität Göttingen, Goldschmidtstr. 1, 37077 Göttingen, Germany

## Abstract

**Background:**

Metagenomics is an approach to the characterization of microbial genomes via the direct isolation of genomic sequences from the environment without prior cultivation. The amount of metagenomic sequence data is growing fast while computational methods for metagenome analysis are still in their infancy. In contrast to genomic sequences of single species, which can usually be assembled and analyzed by many available methods, a large proportion of metagenome data remains as unassembled anonymous sequencing reads. One of the aims of all metagenomic sequencing projects is the identification of novel genes. Short length, for example, Sanger sequencing yields on average 700 bp fragments, and unknown phylogenetic origin of most fragments require approaches to gene prediction that are different from the currently available methods for genomes of single species. In particular, the large size of metagenomic samples requires fast and accurate methods with small numbers of false positive predictions.

**Results:**

We introduce a novel gene prediction algorithm for metagenomic fragments based on a two-stage machine learning approach. In the first stage, we use linear discriminants for monocodon usage, dicodon usage and translation initiation sites to extract features from DNA sequences. In the second stage, an artificial neural network combines these features with open reading frame length and fragment GC-content to compute the probability that this open reading frame encodes a protein. This probability is used for the classification and scoring of gene candidates. With large scale training, our method provides fast single fragment predictions with good sensitivity and specificity on artificially fragmented genomic DNA. Additionally, this method is able to predict translation initiation sites accurately and distinguishes complete from incomplete genes with high reliability.

**Conclusion:**

Large scale machine learning methods are well-suited for gene prediction in metagenomic DNA fragments. In particular, the combination of linear discriminants and neural networks is promising and should be considered for integration into metagenomic analysis pipelines. The data sets can be downloaded from the URL provided (see Availability and requirements section).

## Background

Communities of natural microorganisms often encompass a bewildering range of physiological, metabolic, and genomic diversity. The microbial diversity in most environments exceeds the biodiversity of plants and animals by orders of magnitude. Phylogenetic surveys of complex ecosystems such as soils and sediments have demonstrated that the multitude of discrete prokaryotic species represented in a single sample goes far beyond the number and phenotypes of known cultured microorganisms [1,2]. Direct cultivation or indirect molecular approaches have been used to explore and to exploit this enormous microbial diversity. Cultivation and isolation of microorganisms are the traditional methods. It has been estimated that less than 1 % of environmental microorganisms are culturable using standard cultivation methods. Thus, only a tiny portion of the gene pool of natural microbial communities has been analyzed so far [2-4].

To circumvent some of the limitations of cultivation approaches, indirect molecular methods, such as metagenomics have been developed. Metagenomics is based on the direct isolation, cloning, and subsequent analysis of microbial DNA from environmental samples without prior cultivation [5-7]. Function- and sequence-based analysis of metagenomic DNA fragments have resulted in the identification of a variety of novel genes and gene products [6,8,9]. In addition, partial sequencing of metagenomes, such as those from the acid mine biofilm (75 Mbp) [10], Minnesota farm soil (100 Mbp) [11], and Sargasso Sea (1,600 Mbp) [12], have provided a better understanding of the structure and genomic potential of microbial communities.

A major goal of metagenomic sequencing projects is the identification of protein coding genes. Most genes in metagenomic fragments are currently identified by homology to known genes by employing other methods, e.g. BLAST [13]. The disadvantage of such an approach is obvious: it is impossible to find novel genes that way. Particularly in cases where metagenomic studies aim to discover new proteins, homology search is an inadequate tool for gene prediction.

The computational ab initio prediction of genes from microbial DNA has a long history, and a number of tools have been developed and employed for gene prediction and annotation of genomic sequences from single prokaryotic species (e.g. GLIMMER [14] and GeneMark.hmm [15]). A minor restriction in the application of some conventional approaches to metagenomes is that they are based on the identification of open reading frames (ORFs), which begin with a start codon and end with an in-frame stop codon. Sequenced metagenomes comprise a collection of numerous short sequencing reads of varying length depending on the employed sequencing technique. A typical metagenomic fragment derived by Sanger sequencing [16] is approximately 700 bp long and contains two or fewer genes. The majority of these genes are incomplete, meaning one or both gene ends extend beyond fragment end(s). Therefore, most ORFs in metagenomic sequencing reads will be overlooked by ORF-based gene finders. A more profound problem is that most gene finders for prokaryotic genomes rely on statistical sequence models that are estimated from the analyzed or a closely related genome. Most metagenomic fragments do not bear sufficient sequence information for building statistical models able to distinguish coding from non-coding ORFs. One might consider to derive models from a complete metagenome but the resulting gene prediction quality in fragments from underrepresented species in the metagenome is questionable.

Up to now, there are three approaches for predicting genes from metagenomic DNA fragments. One of these methods is based on BLAST search, where the search is not only applied against databases of known proteins but also against a library constructed from the metagenomic sample itself [17]. In principle, this computationally expensive approach is able to find novel genes, provided that homologues of these genes are contained in the sample. However, it is not clear whether interesting genes will always be conserved in a metagenomic sample. The first method that was developed for ab initio gene prediction in short and anonymous DNA sequences is a heuristic approach of GeneMark.hmm that derives an adapted monocodon usage model from the GC-content of an input sequence [18].

Another method that was developed for ab initio gene prediction in metagenomic DNA fragments is MetaGene [19]. Similar to GeneMark.hmm, MetaGene employs GC-content specific monocodon and dicodon models for predicting genes. The time-efficient two step gene prediction algorithm first extracts ORFs and scores them on the basis of statistical models estimated from fully sequenced and annotated genomes. Subsequently, a dynamic program calculates the final ORF combination from different scores. Additionally, MetaGene utilizes ORF length, the distance from the annotated start codon to the left-most start codon, and distances to neighboring ORFs. Two separate models were estimated from bacterial and archaeal genomes, respectively. The domain specific models are simultaneously applied to each fragment and the higher scoring model is selected for final gene prediction. Results in randomly sampled fragments from annotated genomes indicate that MetaGene provides a high sensitivity in finding genes in fragmented DNA, while the specificity of the predictions is slightly lower. In addition, the performance of GeneMark.hmm in 700 bp fragments and for complete genomes was investigated (supplementary table S3 and table 1 of [19]). Comparable performance results were obtained for both methods for both types of input sequences.

Here, we present a novel approach for gene prediction in single fragments, which is based entirely on machine learning techniques. In bioinformatics, state-of-the-art machine learning methods are usually applied to problems where, at most, several thousands of examples exist for training and evaluation. In our application, learning has to be performed on large data sets with millions of examples. This requires the use of a learning architecture that is capable of large-scale training and testing. Here, we propose a combination of neural networks and linear discriminants. While linear discriminants are used for the extraction of features from high-dimensional data which characterize codon usage and potential gene starts, a small neural network is used for non-linear combination of these features with additional information on length and GC-content of gene candidates. Neural networks in combination with linear discriminants or positional weight matrices have also been applied to other gene prediction problems, for instance in promoter recognition [20].

To provide comparability in our experimental evaluation, we use a setup that is similar to the one used for the initial evaluation of MetaGene. We test our program on fragments from thirteen species. However, we provide some important extensions: We use a higher number of fragments which are randomly sampled from the test genomes to avoid any bias that may result from a particular fragmentation technique. The higher number of fragments is used to cope with the variance across different (repeated) sampling experiments. In addition, we provide a detailed analysis of the translation initiation site (TIS) prediction performance and we also investigate the ability to discriminate between complete and incomplete genes.

## Methods

Most prokaryotic protein coding genes consist of a start codon, followed by a variable number of consecutive in-frame codons and are terminated by a stop codon. This particular arrangement of codons is commonly referred to as open reading frame (ORF). The sole identification of ORFs is not sufficient for prokaryotic gene prediction because the majority of ORFs in a genome are, in fact, non-coding.

In DNA fragments, ORFs frequently exceed the fragment ends. We therefore extend the ORF definition to *incomplete *ORFs.

**Figure 1:**
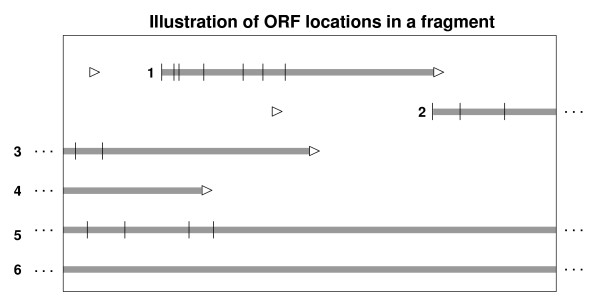
**The figure illustrates possible localizations of open reading frames (ORFs) in a fragment (shown only for the forward strand).** ORFs are shown as grey bars, »▹«denotes stop codons, »|« indicates the position of translation initiation site candidates. ORFs that are related by a common stop codon are grouped and we refer to them as ORF-sets. The box symbolizes the fragment range. Everything that might be located outside the box is invisible to gene prediction algorithms. Further explanations are given in section »Methods«.

The fact that start codons are identical to some *regular *codons results in a high number of related ORFs that share a stop codon but have different start codons. We term such a set of related ORFs an ORF-set and we name the possible start codons of an ORF-set translation initiation site (TIS) candidates. Figure 1 illustrates possible cases of ORF occurrence in a DNA fragment: In case 1, the complete ORF-set is located in the fragment. Additional TIS candidates for this ORF-set can not occur because of an upstream in-frame stop codon. Predicted genes from this ORF-set will always be complete. In case 2, only TIS candidates are located inside the fragment. The range for upstream TIS is again limited by an in-frame stop codon. This candidate, if classified as coding, would result in the prediction of an incomplete gene. In case 3, the stop is located in the fragment. Some TIS candidates are contained in the fragment but there might exist TIS candidates outside the fragment. An ORF-set of this type may result either in a complete or in an incomplete gene. Case 4 is complementary to case 2. Only a stop codon is located inside the fragment. Case 5 and 6 are fragment-spanning ORF-sets, where 5 also includes TIS candidates inside the fragment. Predictions from case 5 will be incomplete but may have a start codon. Case 5 and 6 can both result in the prediction of incomplete genes without start and stop codons.

Our gene prediction algorithm is designed for the discrimination of coding from non-coding ORFs. After the identification of all ORFs in a fragment, we extract features from those ORFs using linear discriminants. Subsequently, we use a neural network that has been particularly trained for the classification of ORFs as coding or non-coding. Classification is based on a gene probability that the neural network assigns to every ORF. Because gene-containing ORF-sets usually comprise of more than one candidate, several ORFs of such an ORF-set may be assigned a high probability by the neural network. The final gene prediction is achieved by a »greedy« method that selects the most probable ORFs that overlap by, at most, 60 bases.

### Machine Learning Techniques

To predict whether a particular ORF actually corresponds to a protein coding region or to a non-coding region, we use a neural network for binary ORF classification. In the following sections, we will first describe the features utilized as inputs for the neural network. Subsequently, we will depict the neural network architecture and the methods we used for large scale training and validation from labeled ORFs in artificial fragments.

#### Features

For realization of the neural network, we use seven features based on sequence characteristics of ORFs. As network inputs, these sequence features are subject to a separate preprocessing step. Below, we explain the methods for computation of these features in detail.

##### Codon and Dicodon Usage

The perhaps most important features for the discrimination between coding and non-coding ORFs can be derived from codon usage, in particular from 4^3 ^monocodon and 4^6 ^dicodon frequencies. These frequencies represent the occurrences of successive trinucleotides (non-overlapping) and hexanucleotides (half-overlapping), respectively. For the characterization of monocodon and dicodon usage, we compute two features based on linear discriminant scores.

Linear discriminants were obtained from training with annotated sequence data. We used coding and non-coding regions from annotated genomes as positive and negative examples, respectively (see section »Training Data for Feature Preprocessing«). Examples are represented by vectors of frequencies of 4^3 ^and 4^6 ^possible monocodons and dicodons, respectively. In the following, we describe discriminant training for the monocodon case. The same training procedure was applied to the dicodon case.

For the *i*-th example, we denote a monocodon frequency vector as $x_{M}^{i}\in {\mathbb{R}}^{64}$, which is the *i*-th column of the data matrix **X**_*M*_, containing all training vectors. To remove length information from these data, all training vectors are normalized to unit Euclidean norm. The corresponding label $y_{M}^{i}$ ∈ {-1, 1}, which is the *i*-th element of the label vector **y**_*M*_, indicates whether the example represents a coding ($y_{M}^{i}$ = 1) or non-coding ($y_{M}^{i}$ = -1) region. For training of the discriminant weight vector **w**_*M*_, we use a regularized least squares approach, i.e. we minimize the following regularized error:

(1) $$E(w_{M})=\frac{1}{n}\sum_{i=1}^{n}{\left(w_{M}\cdot x_{M}^{i}-y_{i}\right)}^{2}+\lambda w_{M}\cdot w_{M}$$

where » · « denotes the dot product. The minimizer of *E *is obtained by [21]:

(2) $$w_{M}={\left(X_{M}X_{M}^{T}+\lambda \;nI\right)}^{-1}X_{M}y_{M}$$

with *d *× *d *identity matrix **I **and with upper *T *and -1 indicating matrix transposition and inversion, respectively. The computational cost scale linearly with the number of examples, which makes the approach well suited for large scale learning. Doing the same for the dicodon frequency discriminant vector **w**_*D*_, we obtain two discriminant scores that serve as the first two input features of the neural network:

(3) *x*_1 _= **w**_*M *_· **x**_*M*_, *x*_2 _= **w**_*D *_· **x**_*D*_.

To adjust the regularization parameter *λ*, we measure the discriminative power of the respective classifier by means of the area under precision recall curve (»auPRC«) as explained in section »Measures of Performance«. Thereby, we choose a *λ *∈ {10^*m*^|*m *= -8, -7, ..., 6} to maximize the auPRC on an independent validation set (see section »Training Data for Feature Preprocessing«).

##### Translation Initiation Site

A discriminant is derived from up- and downstream regions of translation initiation site (TIS) examples. Here we use a 60 basepair (bp) window centered on a potential start codon at window position 31 (see section »Training Data«). We encode the trinucleotide occurrences in that window to yield binary indicator vectors. In each of its 3712 dimensions (64 trinucleotides × 58 positions), a vector indicates whether a certain trinucleotide occurs at a particular window position. Training of the discriminant proceeds in the same way as for the previous two discriminants based on codon usage. Again, we select the regularization parameter *λ *∈ {10^*m*^|*m *= -8, -7, ..., 6} by maximization of the auPRC on an independent validation set.

Because not all genes have a potential TIS region we do not use the TIS score *s *= **w**_*T *_· **x**_*T *_directly, but instead we take the posterior probabilities of being a TIS or not. For computation of the posterior probabilities, we use Gaussian probability density functions of the score:

(4) $$p(s|\mu ,\sigma )=\frac{1}{\sqrt{2\pi }\sigma }\exp(-\frac{1}{2\sigma^{2}}{\left(s-\mu \right)}^{2})$$

where *μ *stands for mean and *σ *for standard deviation.

The features *x*_3 _and *x*_4 _were obtained from a mixture of two Gaussians

(5) *p*(*s*) = *π*^+^*p*(*s*|*μ*^+^, *σ*^+^) + *π*^-^*p*(*s*|*μ*^-^, *σ*^-^)

with parameters estimated from scores of positive and negative training examples, respectively (*π *^+ ^and *π *^- ^are the a priori probabilities of the two classes):

(6) $$\begin{array}{cc} x_{3}=\frac{\pi^{+}p(s|\mu^{+},\sigma^{+})}{p(s)}, & x_{4}=\frac{\pi^{-}p(s|\mu^{-},\sigma^{-})}{p(s)}. \end{array}$$

If no TIS candidate is present, both probabilities are set to zero for that ORF. Note that this case is different from the case of missing values, which can be solved by assigning a priori probabilities for true and false TIS. Here we encounter the possible case where we know that none of the two categories is adequate.

##### Length features

Another feature for discrimination between coding and non-coding ORFs is the sequence length of the ORF. Here, it is important to distinguish between complete and incomplete ORFs. For incomplete ORFs, the observable »incomplete length« is merely a lower bound for the unobservable »complete length« of that ORF and therefore should be treated in a different way. Consequently, we use one »incomplete« and one »complete length« feature. For a particular ORF, only the feature that corresponds to the type of ORF has non-zero value. The value is simply the observed length divided by the maximal length *l*_max_. In our evaluation, we set *l*_max _to 700 bp. In this way we obtain two more features *x*_5_, x_6 _≥ 0 for complete and incomplete length.

##### GC-content

As a last feature *x*_7 _∈ [0, 1], we use, for each ORF, the GC-content estimated from the whole fragment in which this ORF occurs.

#### Neural Network

We use standard multilayer perceptrons with one layer of *k *hidden nodes and with a single logistic output function. Within a binary classification setup with labels *y*_*i *_= 1 (»true«) or *y*_*i *_= 0 (»false«) the output of the neural network can be viewed as an approximation of the posterior probability of the »true« class [22]. In our case, the »true« class represents coding ORFs and therefore the network output can be interpreted in terms of a gene probability. For an input feature vector **x**, the *k *hidden layer activations *z*_*i *_based on input weight vectors $w_{I}^{i}$ and bias parameters $b_{I}^{i}$ are

(7) $$z_{i}=\tanh(w_{I}^{i}\cdot x+b_{I}^{i}).$$

Putting the *z*_*i *_into a vector **z**, the output of the network, i.e. its prediction function based on weight vector **w**_*O *_and bias *b*_*O*_, is

(8) $$g(z)=\frac{1}{1+\exp(-w_{O}\cdot z-b_{O})}.$$

Given a training set **x**_1_, ..., **x**_*N *_and a network with weight and bias parameters collected in the vector ***θ***, we now write the corresponding network output as *f*(**x**_1_; ***θ***), ..., *f*(**x**_*N*_; ***θ***). With diagonal matrix **A **containing the regularization parameters, the training objective is to minimize the regularized error:

(9) $$E(\theta )=\sum_{i=1}^{N}{\left(f(x_{i};\theta )-y_{i}\right)}^{2}+\theta^{T}A\theta .$$

The diagonal matrix **A **= diag(*α*_1_, ..., *α*_1_, *α*_2_, ..., *α*_2_, *α*_3_, ..., *α*_3_, *α*_4_) of the regularization term involves four hyperparameters *α*_1_, *α*_2_, *α*_3_, *α*_4 _> 0 for separate scaling of the parameters $w_{I}^{i}$, $b_{I}^{i}$, **w**_*O*_, *b*_*O*_. Note that the regularization term penalizes the squared magnitude of the weights. For the adaptation of hyperparameters, we utilize the evidence framework [23] based on a Gaussian approximation of the posterior distribution of network weights. The evidence-based adaptation of hyperparameters can be incorporated into the network training procedure and does not require additional validation data. For the minimization of (9) with respect to weight and bias parameters, we use a scaled conjugate gradient scheme, as implemented in the NETLAB toolbox [24]. While weight and bias parameters were initialized randomly according to a standard normal distribution, the hyperparameters were initially set to *α*_1 _= *α*_2 _= *α*_3 _= *α*_4 _= 0.01. The complete training scheme performs 50 iterations where each iteration comprises 50 gradient steps and two successive hyperparameter adaptation steps.

### Final Candidate Selection

Application of the neural network to a certain fragment results in a list of potential gene candidates with a predicted gene probability above 0.5. Most of these predictions are mutually exclusive in terms of overlap. Many predictions even belong to the same ORF-set, differing only in the position of the start codon. In order to obtain a list G of final genes for a particular fragment, predictions with maximal probability are iteratively selected from the list of candidates C, which is successively reduced according to a maximum overlap constraint. Starting with an empty list G and an initial list C containing all fragment-specific ORFs *i *with gene probability *P*_*i *_= *f *(**x**_*i*_; ***θ***) *> *0.5, we apply the following »greedy« selection scheme:

While C is nonempty do

• determine $i_{\max}=\arg\underset{i}{\max}P_{i}$ with respect to all ORFs *i *in C

• remove ORF *i*_max _from C and add it to G

• remove all ORFs from C that overlap with ORF *i*_max _by more than *o*_max _bp

In our evaluation, we set *o*_max _to 60 bp, which corresponds to the minimal gene length we consider for prediction.

### Training Data

Our machine learning approach for gene prediction in metagenomic DNA fragments is based on learning the characteristics of coding and non-coding regions from 131 fully sequenced prokaryotic genomes [see Additional file 1] and their GenBank [25] annotation for protein coding genes. The training genomes correspond to the ones that were used for building the statistical models of MetaGene except that we excluded *Pseudomonas aeruginosa *from the training set because a subset of reliably annotated genes that is valuable for the determination of TIS correctness is available for this species. All training and test data sets described in this article are based on the initial extraction of ORFs with a minimal length of 60 bp. Two types of ORFs are distinguishable: *Complete *ORFs begin with a start codon (ATG, CTG, GTG or TTG), and are followed by a flexible number of subsequent codons and conclude with a stop codon (TAG, TGA or TAA). *Incomplete *ORFs stretch from one fragment end to a stop or start codon or to the other fragment end without being interrupted by another in-frame stop codon (compare Figure 1).

In the following paragraphs, we first describe the preparation of training data sets for feature preprocessing and for training of the neural network. Subsequently, we specify the compilation of a test data set for performance evaluation.

#### Training Data for Feature Preprocessing

Monocodon, dicodon and TIS feature extraction from ORFs require a preprocessing step that is based on the separate training procedure described in section «Codon and Dicodon Usage». Training examples for feature preprocessing were randomly sampled from complete genomes to a coverage of 50 %. Two separate training sets were compiled. For the mono- and dicodon frequencies training set, DNA sequences of genes defined by their exact start and stop codon position served as positive examples (≈1.9 × 10^5^). The longest candidate out of each non-coding ORF-set was selected for the composition of negative examples (≈2.8 × 10^6^).

Training of the TIS discriminant was carried out on symmetric 60 bp sequence windows around start codons. The sequence windows of annotated start codons served as positive examples (1.9 × 10^5^) while the windows around other possible start codons of the same ORF-sets were used as negative examples (5.6 × 10^6^).

The examples for both training data sets were randomly split into 50 % for discriminant training and 50 % for validation of the regularization parameter.

#### Training Data for the Neural Network

The neural network was trained with the extracted features from ORFs in 700 bp fragments that were randomly excised to a 1-fold genome coverage from each training genome. We define an n-fold coverage as the amount of sampled DNA that is in total length (bp) n times longer than the original genome sequence. Annotated genes in these fragments were used as positive examples for coding regions (≈2.6 × 10^6^) while one candidate out of each non-coding ORF-set was randomly selected for the negative examples (≈4.5 × 10^6^). The data sets were randomly split into 50% for neural network training and 50 % for validation of the network size (see section »Neural Network«).

### Test Data and Experimental Evaluation

The performance of our gene prediction algorithm was evaluated on artificial DNA fragments from three archaeal and ten bacterial species (see Table 1) whose genera were not used for training. Fragments of the lengths 100 to 2000 bp (in intervals of 100 bp) were randomly sampled from each genome to a 5-fold genome coverage for each length. We used the fragments of all lengths to investigate gene prediction performance of our method, which was trained on fragments with the length 700 bp.

**Table 1:** Genomes of microbial species that were used for the evaluation of our method. The upper three species are archaea while the lower ten species belong to the bacterial domain. The table shows GenBank accession numbers (GenBank Acc.), and genome sizes (Size).

Species	GenBank Acc.	Size (Mbp)
*Archaeoglobus fulgidus*	NC_000917	2.2
*Methanococcus jannaschii*	NC_000909	1.7
*Natronomonas pharaonis*	NC_007426	2.6

*Buchnera aphidicola*	NC_002528	0.6
*Burkholderia pseudomallei*	NC_006350, NC_006351	7.2
*Bacillus subtilis*	NC_000964	4.2
*Corynebacterium jeikeium*	NC_007164	2.5
*Chlorobium tepidum*	NC_002932	2.2
*Escherichia coli*	NC_000913	4.6
*Helicobacter pylori*	NC_000921	1.6
*Pseudomonas aeruginosa*	NC_002516	6.3
*Prochlorococcus marinus*	NC_007577	1.7
*Wolbachia endosymbiont*	NC_006833	1.1

A more detailed analysis was carried out on 700 bp fragments (also sampled to a 5-fold coverage), including a comparison to MetaGene. In order to determine statistical significance, we used 10 replicates of each randomly sampled fragment stack.

Gene prediction performance was evaluated by comparing predictions of our method to known annotated genes in fragments. The GenBank annotation for protein coding genes was used to measure general gene prediction performance. However, the GenBank gene start annotation has previously been suspected to be inaccurate [26]. Therefore, we used »reliable gene annotation subsets« [27] for the evaluation of translation initiation site (TIS) prediction performance: all genes with an experimentally verified TIS from »EcoGene« for *Escherichia coli *[28], experimentally verified genes of the *Bacillus subtilis *GenBank annotation (all non-y genes) and the »PseudoCAP« (Pseudomonas community annotation project) annotation of *Pseudomonas aeruginosa *[29].

#### Measures of Performance

The capability of detecting annotated genes (and genes including their annotated TIS) was measured as sensitivity:

(10) $$\text{Sens}=\frac{\text{TP}}{\text{TP}+\text{FN}}$$

For gene prediction sensitivity, TP_gene _(true positives) denotes correct matches and FN_gene _(false negatives) indicate overlooked genes. We counted all predictions as TP_gene _that match at least 60 bp in the same reading-frame to an annotated gene.

In one experiment, we compared gene predictions to a subset of genes that have a reliably annotated gene start in the fragment. For this subset, we measured TIS prediction sensitivity. Here, TP_TIS _are genes with correctly predicted TIS and FN_TIS _are genes whose correct start codons were not predicted.

The reliability of gene predictions was measured by specificity:

(11) $$\text{Spec}=\frac{{\text{TP}}_{\text{gene}}}{{\text{TP}}_{\text{gene}}+{\text{FP}}_{\text{gene}}}$$

Gene prediction specificity was calculated with predicted genes that do not correspond to any gene in the annotation as FP_gene _(false positives).

To provide a suitable composite measure of sensitivity and specificity we use the harmonic mean, which corresponds to a particular realization of the F-measure [30]:

(12) $$\text{HarmonicMean}=\frac{2^{*}{\text{Sens}}^{*}\text{Spec}}{\text{Sens}+\text{Spec}}.$$

To measure the discriminative power of the codon usage and TIS discriminants for feature extraction (see »Features«), we use the area under *precision recall curve *(auPRC). The precision recall curve shows for each possible score threshold the relation of sensitivity (on the x-axes) and specificity (on the y-axes). Sensitivity and specificity are not sufficient for measuring TIS prediction performance. When applied to TIS prediction, these measures rather reflect general gene prediction performance than accuracy of TIS prediction. 'TIS correctness' was therefore measured by the percentage of correctly predicted TIS within a subset of true positive gene predictions TP_gene _that have an annotated start codon within the fragment (TP_gene _that have annotated start codon wihin the fragment (TP_gene_*):

(13) $$\text{TIS correctness}=\frac{{\text{TP}}_{\text{TIS}}}{{\text{TP}}_{{\text{gene}}^{*}}}.$$

Accuracy of complete/incomplete gene type prediction was calculated on the basis of correctly predicted genes with an existing true TIS:

(14) $$\text{GeneTypeAccuracy}=\frac{{\text{TP}}_{\text{complete}}+{\text{TN}}_{\text{complete}}}{{\text{TP}}_{\text{gene}}}$$

where TP_complete _and TN_complete _account for the number of genes within TP_gene _that have correctly been predicted as complete and incomplete.

## Results and Discussion

In the following sections, we first describe and discuss the results of discriminant and neural network validation which led to the choice of a hyperparameter *λ *and a suitable number of nodes for the neural net. Subsequently, we show and discuss gene prediction performance results of the neural network on several fragment lengths and in 700 bp fragments.

### Discriminant Validation

Training of the linear discriminants for monocodon, dicodon and TIS features requires the validation of the regularization parameter *λ *. For each of the three discriminants, we chose *λ *from the set of values {10^*m*^|*m *= -8, -7, ..., 6} by maximizing the area under precision recall curve (auPRC) on separate validation data. While for the TIS discriminant, a well-defined maximum was achieved for an intermediate *λ *= 10^-2^, for the monocodon and dicodon case the maximum was achieved for the smallest value *λ *= 10^-8^. However, as shown in Additional file 2, for small *λ *values the auPRC performance in these cases reaches a plateau and therefore we did not try smaller values. The resulting discriminant weights for the 64 monocodons are shown in Additional file 1. The high negative weights for the three stop codons TAA, TAG, TGA are due to the large fraction of negative examples. Because negative examples are, by a factor 10, more frequent than positive examples in the training set, a negative shift of the discriminant score is induced by codons that, like stop codons, are present in any example in any of the two classes.

### Network Validation

In principle, the evidence-based hyperparameter adaptation (see section »Neural Network«) obviates the search for an adequate size of the network, i.e. to find a suitable number *k *of hidden nodes. The network size has just to be large enough to provide maximum performance, while larger nets would automatically be subject to stronger regularization in terms of larger regularization parameters. Nevertheless, network size is crucial in terms of computational cost for training and testing.

In order to find a small network with sufficient performance, we started to train networks of increasing size. Trying networks with *k *= 5, 10, ..., 25 nodes, we found the performance to reach a nearly flat plateau within that *k*-range, with only very slight increase above *k *= 15 [see Additional file 2]. Performance was measured in terms of the harmonic mean criterion (see section »Measures of Performance«), computed on an independent validation set (see section »Training Data«). For the final predictions on the test data, we used the largest network with *k *= 25 nodes.

While training of neural networks is a time consuming process, computing predictions with a trained network on new data is very fast. In our case, training a network with *k *= 25 hidden nodes from ≅ 3.6 × 10^6 ^examples took about 190 cpu hours (AMD Opteron, 2 GHz). The training scheme described above was applied in parallel to 5 networks with different (random) initialization of parameters to avoid weak local minima of the regularized error (9). According to the lowest error, the best resulting network was selected for the final predictions within the test setup. In contrast, testing of the same number of examples, i.e. prediction on more than three million candidates, only took ≅ 16.5 seconds on the same machine.

The parameters of the neural network with 25 nodes that we used for further evaluation are given in Additional file 1.

### Gene Prediction Performance in DNA fragments

To evaluate the performance of our machine learning approach, we tested the method on artificially fragmented genomes. In the following section, we present the results in general gene prediction performance on various fragment lengths. Subsequently, we analyze gene prediction performance, TIS prediction correctness and complete/incomplete gene type prediction accuracy in detail for fragments of length 700 bp, which corresponds to the fragment length on which the neural network was trained.

#### Performance in Fragments of Different Lengths

**Figure 2:**
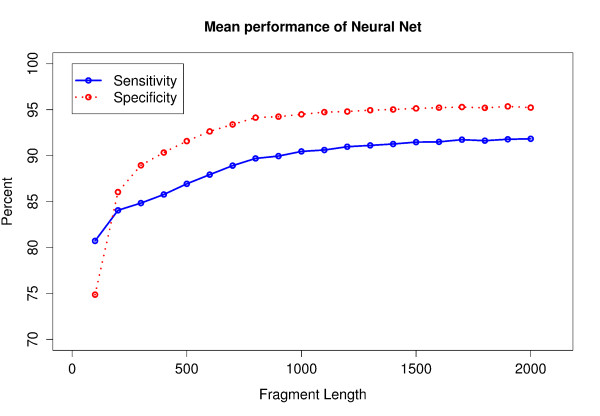
**Average gene prediction performance of the neural network in fragments of the lengths 100 to 2000 bp.** The performance values from thirteen test species were averaged by arithmetic mean.

The predictions of our method in DNA fragments with lengths ranging from 100 to 2000 bp from thirteen species were compared to the GenBank [25] annotation for protein coding genes. Note that on average 15 % of 100 bp fragments do not contain any annotated gene matching the 60 bp minimal length criterion (complete or incomplete), for 700 bp fragments, this fraction of fragments accounts 3 %, for 2000 bp fragments 0.8 %. The average percentage of complete genes within all annotated genes in our test fragments is 0 % for 100 bp fragments, 8 % for 700 bp fragments and 40 % for 2000 bp fragments [see Additional file 2]. The mean of gene prediction sensitivity and specificity for all fragment lengths is shown in Figure 2. On 700 bp fragments, our method has an average gene prediction sensitivity of 89 % and an average specificity of 93 %. Sensitivity and specificity slightly increase with growing fragment size. This can be explained by the fact that ORFs carrying distinct mono-/dicodon and TIS signals occur more often in longer fragments. Gene prediction performance decreases with length and sharply drops for fragments shorter than 200 bp.

Before application of our method on real metagenomes, the neural network should be trained and thoroughly evaluated on fragments of a length that corresponds to the real fragments of interest. Metagenomic sequencing projects differ in their aims and in the applied sequencing and annotation strategies. Improved Sanger sequencing from one or both vector insert ends is applied in many metagenomic projects (examples are listed in review [31]) and yields sequencing reads roughly ranging from 500 to 1000 bp. Based on the current results, our method might be particularly useful for improving gene annotation and discovery on Sanger sequencing reads. However, pyrosequencing [32] has also been introduced to metagenomics [33]. The pyrosequencing approach does not involve any cloning step. With recent improvements, pyrosequencing now yields a read length between 200 and 300 bp [34]. In principle, it should be possible to predict genes in such short fragments with our fragment-based techniques but environmental pyrosequencing projects may rather be focused on phylogenetic studies and habitat comparison than on the discovery of new genes. In some metagenomic sequencing projects, long metagenomic inserts (up to 40 kbp) are fully sequenced [35]. Although gene prediction performance of the neural network does not decrease on longer fragments [see Additional file 2], other methods like MetaGene [19] or GeneMark.hmm [18], which also consider the context of putative genes (e.g. operons), may be more suitable for fragments of this size.

#### Performance in 700 bp Fragments

Predicted genes in fragments with a length of 700 bp from three archaeal and ten bacterial species were compared to the GenBank annotation for protein coding genes. The mean and standard deviation for sensitivity, specificity and the harmonic mean of 10 repetitions per species are shown in Table 2. The neural network has high sensitivity (ranging from 82 to 92 %) and specificity (ranging from 85 to 97 %) in fragments from all species. We could not observe a major performance difference for sensitivity and specificity between archaeal and bacterial fragments but the variation between different species in general is large.

**Table 2:** Mean and standard deviation for gene prediction performance of our method (Neural Net) and MetaGene. Performance was measured on 700 bp fragments that were randomly excised from each test genome to 5-fold coverage (ten replications per species). The harmonic mean is a measure that combines sensitivity and specificity.

	SENSITIVITY		SPECIFICITY		HARMONIC MEAN	
Species	Neural Net	MetaGene	Neural Net	MetaGene	Neural Net	MetaGene
*Archaeoglobus fulgidus*	87.2 ± 0.21	**93.7 **± **0.15**	**93.4 **± **0.16**	92.7 ± 0.16	90.2 ± 0.17	**93.2 **± **0.14**
*Methanococcus jannaschii*	91.7 ± 0.17	**95.8 **± **0.14**	**96.2 **± **0.13**	92.7 ± 0.19	93.9 ± 0.10	**94.3 **± **0.15**
*Natronomonas pharaonis*	87.9 ± 0.22	**95.1 **± **0.09**	**93.9 **± **0.10**	92.7 ± 0.17	90.8 ± 0.16	**93.9 **± **0.12**
*Buchnera aphidicola*	90.6 ± 0.37	**96.7 **± **0.24**	**95.3 **± **0.31**	91.1 ± 0.29	92.9 ± 0.28	**93.8 **± **0.21**
*Burkholderia pseudomallei*	87.9 ± 0.11	**94.1 **± **0.11**	**90.1 **± **0.09**	85.1 ± 0.13	89.0± 0.08	**89.4 **± **0.10**
*Bacillus subtilis*	**91.4 **± **0.16**	89.8 ± 0.14	**95.3 **± **0.09**	89.3 ± 0.19	**93.3 **± **0.10**	89.5 ± 0.14
*Corynebacterium jeikeium*	89.7 ± 0.24	**91.9 **± **0.12**	**93.8 **± **0.19**	89.2 ± 0.21	**91.7 **± **0.19**	90.5 ± 0.13
*Chlorobium tepidum*	82.1 ± 0.25	**85.7 **± **0.27**	**91.2 **± **0.17**	88.4 ± 0.26	86.4 ± 0.19	**87.0 **± **0.22**
*Escherichia coli*	91.7 ± 0.16	**93.3 **± **0.07**	**95.3 **± **0.09**	90.9 ± 0.10	**93.5 **± **0.12**	92.1 ± 0.07
*Helicobacter pylori*	**92.1 **± **0.11**	90.2 ± 0.14	**96.6 **± **0.15**	89.6 ± 0.23	**94.3 **± **0.11**	89.9 ± 0.15
*Pseudomonas aeruginosa*	90.4 ± 0.14	**96.2 **± **0.07**	**92.5 **± **0.11**	91.4 ± 0.09	91.4 ± 0.12	**93.7 **± **0.07**
*Prochlorococcus marinus*	87.2 ± 0.21	**93.7 **± **0.25**	**95.9 **± **0.14**	90.8 ± 0.20	91.4 ± 0.15	**92.2 **± **0.19**
*Wolbachia endosymbiont*	87.2 ± 0.27	**90.6 **± **0.42**	**85.2 **± **0.44**	71.2 ± 0.54	**86.2 **± **0.29**	79.7 ± 0.45

In comparison to MetaGene, the neural network has a higher specificity in fragments from all test species (on average 4.6 % higher). On the other hand, MetaGene has a higher sensitivity in fragments from most species (on average 3.8 % higher). The neural network only shows a higher sensitivity in *Bacillus subtilis *and *Helicobacter pylori*. The overall performance of both methods calculated by harmonic mean is very similar. For some species, the neural network yields a better overall gene prediction performance while MetaGene performs better on other species. In particular, MetaGene performs better in all tested archaea. All local pairwise differences in sensitivity, specificity and harmonic mean between the neural network and MetaGene are significant to a confidence level of 95 % according to Wilcoxon's signed rank test [36] (R-package *exactRankTests *[37]).

A precise TIS prediction is very important in metagenomics since the aim of many environmental sequencing projects is the identification and subsequent experimental investigation of novel genes. For example, the expression of a metagenomic protein in a host organism may fail or yield incorrect results if the predicted start codon is incorrect. Accurate TIS prediction is a difficult task, even for conventional gene finders on complete genomes [38-42]. This is because ATG, CTG, GTG and TTG also occur inside genes.

One gene may for example contain several ATGs but only one corresponds to a TIS. Our approach includes a TIS-model that is based on a linear discriminant. We measured TIS prediction performance of our algorithm for all correctly predicted genes that have annotated start codons within a fragment. First, we investigated TIS performance on our complete set of test species fragments according to GenBank annotation. The results are shown in Table 3. TIS correctness of our algorithm varies remarkably between different test species. On some bacterial species, our algorithm reaches a TIS correctness of 87 % (e.g. in *Helicobacter pylori*). The lowest TIS performance can be observed in fragments from the bacterium *Chlorobium tepidum *(68 %). The average TIS correctness of our algorithm is around 78 %. In comparison to this, the highest performance of MetaGene can be observed for fragments of *Prochlorococcus marinus *(89 %), the lowest for fragments of *Bacillus subtilis *(66 %). Note that TIS correctness depends on the number of correctly predicted genes with an annotated TIS. Therefore, TIS correctness of our algorithm is not directly comparable to the one obtained by MetaGene, which detects a higher number of genes. However, the variation in TIS correctness of both methods is large.

**Table 3:** Translation initiation site prediction correctness (TIS correctness) and complete/incomplete classifi-cation accuracy (Gene Type Accuracy) of the Neural Net and MetaGene according to GenBank annotation. Performance was measured on 700 bp fragments that were randomly excised from each test genome to 5-fold coverage (mean and standard deviation for 10 replicates per species are given).

	TIS CORRECTNESS		GENE TYPE ACCURACY	
Species	Neural Net	MetaGene	Neural Net	MetaGene
*Archaeoglobus fulgidus*	69.8 ± 0.32	73.6 ± 0.32	98.1 ± 0.05	97.2 ± 0.07
*Methanococcus jannaschii*	69.4 ± 0.52	73.3 ± 0.52	99.0 ± 0.09	97.6 ± 0.12
*Natronomonas pharaonis*	75.2 ± 0.58	82.9 ± 0.28	96.9 ± 0.16	97.6 ± 0.09
*Buchnera aphidicola*	86.5 ± 0.40	88.6 ± 0.64	99.1 ± 0.09	98.3 ± 0.21
*Burkholderia pseudomallei*	70.1 ± 0.45	73.0 ± 0.28	97.6 ± 0.08	96.9 ± 0.09
*Bacillus subtilis*	79.7 ± 0.32	66.1 ± 0.42	98.6 ± 0.05	97.0 ± 0.08
*Corynebacterium jeikeium*	78.2 ± 0.49	73.4 ± 0.68	98.1 ± 0.08	96.6 ± 0.11
*Chlorobium tepidum*	68.1 ± 0.46	71.9 ± 0.45	98.1 ± 0.08	96.7 ± 0.13
*Echerichia coli*	84.5 ± 0.31	78.2 ± 0.15	98.7 ± 0.06	97.0 ± 0.08
*Helicobacter pylori*	87.3 ± 0.40	77.1 ± 0.33	99.2 ± 0.09	96.4 ± 0.16
*Pseudomonas aeruginosa*	78.4 ± 0.22	81.0 ± 0.36	97.7 ± 0.03	97.2 ± 0.07
*Prochlorococcus marinus*	86.6 ± 0.40	88.6 ± 0.47	99.0 ± 0.07	97.8 ± 0.10
*Wolbachia endosymbiont*	79.3 ± 0.77	79.9 ± 0.42	98.7 ± 0.13	96.9 ± 0.17

A reason for this variation might be that the GenBank gene annotation contains many hypothetical and not experimentally verified genes [26]. Therefore, we also evaluated TIS prediction performance on »reliable annotation subsets« of the bacteria *Escherichia coli*, *Bacillus subtilis *and *Pseudomonas aeruginosa *(see section »Test Data and Experimental Evaluation«). Evaluating gene prediction performance in fragments according to these annotation subsets, our algorithm achieves a highly consistent TIS prediction performance between 81 and 87 % in fragments from all three test species. The TIS prediction sensitivity varies from 68 % to 80 % (see Table 4). In comparison, MetaGene's TIS performance shows a higher variation, ranging from 70 to 84 % while the TIS sensitivity ranges from 62 to 80 %.

**Table 4:** Translation initiation site prediction performance of the new gene prediction algorithm (Neural Net) and MetaGene according to »reliable annotation subsets« (A subset of »verified genes« from »EcoGene« for *Escherichia coli *[28], all non-y genes of the *Bacillus subtilis *GenBank annotation and the »PseudoCAP« annotation of *Pseudomonas aeruginosa *[29]). TIS prediction sensitivity and correctness were measured on artificial 700 bp fragments that were randomly excised from each test genome to 5-fold coverage. Mean and standard deviation over 10 replicates per species are shown.

	SENSITIVITY TIS		TIS CORRECTNESS	
Species	Neural Net	MetaGene	Neural Net	MetaGene
*Bacillus subtilis*	**73.4 **± **1.79**	62.1 ± 1.43	84.1 ± 0.51	70.2 ± 0.64
*Escherichia coli*	**80.0 **± **0.68**	75.1 ± 0.61	86.6 ± 0.57	77.5 ± 0.67
*Pseudomonas aeruginosa*	68.0 ± 0.22	**79.7 **± **0.44**	80.7 ± 0.20	83.7 ± 0.36

The nature of fragmented DNA results in the occurrence of complete and incomplete genes. A gene may be incorrectly predicted as complete or incomplete if it has several TIS candidates of which at least one is located outside the fragment. Due to the short fragment length of 700 bp, the vast majority of annotated genes (≈90 %) in our test fragments is incomplete. The experimental strategy of many metagenomic projects relies on sequencing the fragment from one or both ends of the vector insert. Although the insert is not always sequenced completely, sequencing of the entire fragment is possible in case the biologist is interested in further analysis of an incomplete gene. Therefore, it is important to know whether a gene is contained in a sequencing read completely or incompletely.

We evaluated the percentage of genes that were correctly classified as complete or incomplete within the correctly identified genes according to GenBank annotation. Our method achieves an average accuracy of 98 % with little variation (see Table 3). It can be noted that MetaGene slightly more often misclassifies genes concerning their completeness. Note here that the performance indices of the neural network and MetaGene in Table 3 are not directly comparable because they rely on different numbers of correctly identified genes.

#### Remarks on the Experimental Setup

The evaluation of computational methods for metagenomic gene prediction is troubled by the fact that reliably annotated metagenomes are not available. Some metagenomes have been subject to annotation for several years by now, but their gene annotation is far from complete. Particularly, the exact location of gene starts on metagenomes has been verified experimentally only in rare cases. Currently, the only way to reliably investigate gene prediction accuracy is the evaluation on DNA fragments from complete microbial genomes. For the evaluation of our method, we used an experimental setup similar to the one proposed by the authors of MetaGene in order to keep both methods comparable. MetaGene relies on statistical models built from 116 bacterial and 15 archaeal genomes. These species were selected to represent every genus from GenBank in the year 2006. By now, species belonging to many additional genera have been fully sequenced and annotated. Members of these genera should be included in the training set of a future gene prediction tool version in order to collect as much information about the characteristics of coding and non-coding ORFs as possible.

It remains an open question, which criteria are most suitable for the selection of training species. In general, taxonomy does not reflect phylogeny properly. Some species of different genera for example exhibit highly similar codon usage patterns. Particularly for the identification of novel genes in metagenomes whose biological diversity is yet unknown, the transfer of the GenBank bias toward single species should be avoided in the training data set. To reduce this bias, training genomes could be selected according to other criteria, e.g. GC-content, oligonucleotide frequencies or monocodon/dicodon frequencies in protein coding regions.

The experimental setup chosen here also differs from real metagenomes with respect to sequencing errors. The effect of sequencing errors in terms of base-changes on gene prediction performance of our method would depend on the frequency of such kind of error. The effect of a usually small number of base-errors (less than one error per 10 000 bp after routine fragment end removal [19]) can be neglected. As for other alignment-free methods, like MetaGene, our method is susceptible to frame shifts. Only certain alignment-based methods can be expected to be more robust with regard to this kind of error [17].

## Conclusion

Large scale machine learning is well suitable for gene prediction in metagenomic DNA fragments. Due to performance results obtained with the current experimental setup, we suggest that our machine learning approach, with its high gene prediction specificity, TIS correctness and complete/incomplete prediction capabilities, complements MetaGene with its high gene finding sensitivity well. Thus, a combination of both methods should be considered.

## Software availability

Linear discriminants and the trained neural network are available as MATLAB files for download at [43]. A command line tool for gene prediction in DNA fragments (Linux, 64-bit architecture) is available from the authors on request.

## Availability and requirements

Orphelia: 

## Authors' contributions

KJH developed and implemented the combination of ORF extraction and machine learning modules, assembled training and test data sets and performed the evaluation of the new algorithm and MetaGene. MT developed and implemented ORF-set identification and extraction. KJH and MT contributed biological expertise, T.L. implemented fast versions of the discriminant scoring. RD contributed biological expertise on metagenomics and drafted parts of the manuscript. PM contributed machine learning expertise and designed and implemented the machine learning architecture. BM and TL supported the project and contributed conceptually. KJH and PM wrote the manuscript. All authors read and approved the manuscript.

## Supplementary Material

Additional file 1 **Tables with training genomes, discriminant weights, and network parameters**. The tables list all genomes that were used for training the neural network (1), present the discriminant weights that were learned for all monocodons (2), and give neural network parameters (3, 4). Click here for file

Additional file 2 **Supplementary figures**. The figures show the area under precision recall curve for discriminant validation using different *λ *values (1), the neural network performance with increasing numbers of nodes (2), the percentage of complete genes within all annotated genes per fragment for different fragment lengths (3), and gene prediction performance on fragments ranging from 5000 to 60000 bp (4). Click here for file
