# Supplementary material for: Gene prediction in metagenomic fragments: A large scale machine learning approach
Source: BMC Bioinformatics. 2008 Apr 28;9:217. doi: 10.1186/1471-2105-9-217 (PMC2409338; doi:10.1186/1471-2105-9-217)
Supplement: Additional file 1 — Tables with training genomes, discriminant weights, and network parameters. The tables list all genomes that were used for training the neural network (1), present the discriminant weights that were learned for all monocodons (2), and give neural network parameters (3, 4). [file 1471-2105-9-217-S1.pdf]

Additional file 1 — Tables with training genomes, discriminant weights, and network parameters
Supplementary Table 1

Species whose genomes were used for training linear discriminants and neural networks. Fragments excised from these genomes were used for training the neural network.

Species	GenBank accession number(s)
Acinetobacter sp ADP1	NC_005966
Agrobacterium tumefaciens C58 UWash	NC_003304, NC_003305
Anabaena variabilis ATCC 29413	NC_007413
Anaplasma marginale St Maries	NC_004842
Aquifex aeolicus	NC_000918
Azoarcus sp EbN1	NC_006513
Bacteroides fragilis NCTC 9434	NC_003228
Bartonella henselae Houston-1	NC_005956
Bdellovibrio bacteriovorus	NC_005363
Bifidobacterium longum	NC_004307
Bordetella bronchiseptica	NC_002927
Bradyrhizobium japonicum	NC_004463
Brucella abortus 9-941	NC_006932, NC_006933
Campylobacter jejuni RM1221	NC_003912
Carboxydothermus hydrogenoformans Z-2901	NC_007503
Caulobacter crescentus	NC_002696
Chlamydia muridarum	NC_002620
Chlamydophila abortus S26 3	NC_004552
Chromobacterium violaceum	NC_005085
Clostridium acetobutylicum	NC_003030
Colwellia psychrerythraea 34H	NC_003910
Coxiella burnetii	NC_002971
Cyanobacteria bacterium Yellowstone A-Prime	NC_007775
Dechloromonas aromatica RCB	NC_007298
Dehalococcoides ethenogenes 195	NC_002936
Deinococcus radiodurans	NC_001263, NC_001264
Desulfotalea psychrophila LSv54	NC_006138
Desulfovibrio desulfuricans G20	NC_007519
Ehrlichia canis Jake	NC_007354
Enterococcus faecalis V583	NC_004668
Erwinia carotovora atroseptica SCRI1043	NC_004547
Erythrobacter litoralis HTCC2594	NC_007722
Francisella tularensis tularensis	NC_006570
Frankia CcI3	NC_007777
Geobacillus kaustophilus HTA426	NC_006510
Geobacter metallireducens GS-15	NC_007517
Gloeobacter violaceus	NC_005125
Gluconobacter oxydans 621H	NC_006677

Haemophilus ducreyi 35000HP	NC_002940
Hahella chejuensis KCTC 2396	NC_007645
Idiomarina loihiensis L2TR	NC_006512
Lactobacillus acidophilus NCFM	NC_006814
Lactococcus lactis	NC_002662
Legionella pneumophila Lens	NC_006369
Leifsonia xyli xyli CTCB0	NC_006087
Leptospira interrogans serovar Copenhageni	NC_005823, NC_005824
Listeria innocua	NC_003212
Magnetospirillum magneticum AMB-1	NC_007626
Mannheimia succiniciproducens MBEL55E	NC_006300
Mesorhizobium loti	NC_002678
Methylococcus capsulatus Bath	NC_002977
Moorella thermoacetica ATCC 39073	NC_007644
Mycobacterium avium paratuberculosis	NC_002944
Neisseria gonorrhoeae FA 1090	NC_002946
Nitrobacter winogradskyi Nb-255	NC_007406
Nitrosococcus oceani ATCC 19707	NC_007484
Nitrosomonas europaea	NC_004757
Nitrosospira multiformis ATCC 25196	NC_007614
Nocardia farcinica IFM10152	NC_006361
Nostoc sp	NC_003272
Novosphingobium aromaticivorans DSM 12444	NC_007794
Oceanobacillus iheyensis	NC_004193
Onion yellows phytoplasma	NC_005303
Pasteurella multocida	NC_002663
Pelobacter carbinolicus	NC_007498
Pelodictyon luteolum DSM 273	NC_007512
Photobacterium profundum SS9	NC_006370, NC_006371
Photorhabdus luminescens	NC_005126
Porphyromonas gingivalis W83	NC_002950
Propionibacterium acnes KPA171202	NC_006085
Pseudoalteromonas haloplanktis TAC125	NC_007481, NC_007482
Psychrobacter arcticum 273-4	NC_007204
Ralstonia eutropha JMP134	NC_007348, NC_007347
Rhizobium etli CFN 42	NC_007761
Rhodobacter sphaeroides 2 4 1	NC_007493, NC_007494
Pirellula sp	NC_005027
Rhodopseudomonas palustris CGA009	NC_005296
Rhodospirillum rubrum ATCC 11170	NC_007643
Rickettsia conorii	NC_003103
Salinibacter ruber DSM 13855	NC_007677
Salmonella enterica Choleraesuis	NC_006905
Shewanella oneidensis	NC_004347
Shigella boydii Sb227	NC_007613
Silicibacter pomeroyi DSS-3	NC_003911

Sinorhizobium meliloti	NC_003047
Sodalis glossinidius morsitans	NC_007712
Staphylococcus aureus RF122	NC_007622
Streptococcus agalactiae 2603	NC_004116
Streptomyces avermitilis	NC_003155
Symbiobacterium thermophilum IAM14863	NC_006177
Synechococcus elongatus PCC 6301	NC_006576
Synechocystis PCC6803	NC_000911
Thermoanaerobacter tengcongensis	NC_003869
Thermobifida fusca YX	NC_007333
Thermosynechococcus elongatus	NC_004113
Thermotoga maritima	NC_000853
Thermus thermophilus HB27	NC_005835
Thiobacillus denitrificans ATCC 25259	NC_007404
Thiomicrospira crunogena XCL-2	NC_007520
Treponema denticola ATCC 35405	NC_002967
Tropheryma whipplei TW08 27	NC_004551
Vibrio cholerae	NC_002505, NC_002506
Wolinella succinogenes	NC_005090
Xanthomonas citri	NC_003919
Xylella fastidiosa	NC_002488
Yersinia pestis CO92	NC_003143
Zymomonas mobilis ZM4	NC_006526
Aster yellows witches-broom phytoplasma AYWB	NC_007716
Borrelia burgdorferi	NC_001318
Candidatus Blochmannia floridanus	NC_005061
Fusobacterium nucleatum	NC_003454
Mesoplasma florum L1	NC_006055
Mycoplasma capricolum ATCC 27343	NC_007633
Ureaplasma urealyticum	NC_002162
Wigglesworthia brevipalpis	NC_004344
Aeropyrum pernix	NC_000854
Haloarcula marismortui ATCC 43049	NC_006396, NC_006397
Halobacterium sp	NC_002607
Methanococcus maripaludis S2	NC_005791
Methanopyrus kandleri	NC_003551
Methanosarcina acetivorans	NC_003552
Methanobacterium thermoautotrophicum	NC_000916
Nanoarchaeum equitans	NC_005213
Picrophilus torridus DSM 9790	NC_005877
Pyrobaculum aerophilum	NC_003364
Pyrococcus abyssi	NC_000868
Sulfolobus acidocaldarius DSM 639	NC_007181
Thermococcus kodakaraensis KOD1	NC_006624
Thermoplasma acidophilum	NC_002578

Supplementary Table 2

Discriminant weights that were learned for all monocodons.

Codon	Weight
AAA	0.060
AAC	0.028
AAG	0.072
AAT	-0.080
ACA	-0.035
ACC	0.097
ACG	-0.115
ACT	-0.018
AGA	-0.230
AGC	-0.275
AGG	-0.386
AGT	-0.071
ATA	-0.371
ATC	0.115
ATG	0.092
ATT	0.174
CAA	-0.031
CAC	-0.520
CAG	-0.342
CAT	-0.379
CCA	-0.308
CCC	-0.194
CCG	-0.232
CCT	-0.142
CGA	-0.428
CGC	-0.175
CGG	-0.369
CGT	-0.091
CTA	-0.107
CTC	-0.105
CTG	0.365
CTT	-0.265
GAA	0.694
GAC	0.431
GAG	0.596

GAT	0.175
GCA	0.083
GCC	0.047
GCG	-0.095
GCT	-0.106
GGA	-0.092
GGC	-0.062
GGG	-0.160
GGT	-0.117
GTA	-0.234
GTC	-0.116
GTG	0.278
GTT	-0.110
TAA	-4.863
TAC	0.382
TAG	-4.977
TAT	0.225
TCA	-0.178
TCC	-0.303
TCG	-0.288
TCT	-0.165
TGA	-4.550
TGC	-0.361
TGG	0.079
TGT	-0.254
TTA	0.091
TTC	-0.230
TTG	-0.222
TTT	-0.216

Supplementary Table 3

Neural network parameters as described in section 2.1.2.

	$\mathbf{w}_I^1$	$\mathbf{w}_I^2$	$\mathbf{w}_I^3$	$\mathbf{w}_I^4$	$\mathbf{w}_I^5$	$\mathbf{w}_I^6$	$\mathbf{w}_I^7$	$\mathbf{b}_I$	$\mathbf{w}_O$
Node 1	-2.325	1.887	0.031	-0.422	-0.932	-1.292	-1.625	1.056	-2.645
Node 2	-0.652	-0.100	20.850	-5.932	-0.413	-0.347	-0.918	7.473	10.492
Node 3	0.062	-0.687	-0.112	-0.193	-0.585	8.110	1.088	-0.294	4.867
Node 4	0.456	-1.258	-0.922	-0.536	7.129	-0.675	1.218	-0.759	3.368
Node 5	-1.896	2.782	0.472	0.307	0.592	-0.948	-1.294	1.172	3.851
Node 6	1.312	0.366	-0.523	-0.482	1.794	1.060	-2.533	3.563	4.358
Node 7	-1.165	1.419	1.989	0.630	-0.588	2.474	-0.474	1.022	-3.640
Node 8	-0.545	-0.168	-0.295	0.215	-0.143	-0.914	-0.027	-0.719	-0.608
Node 9	-0.869	-2.076	0.310	0.522	1.517	1.685	0.041	-0.902	2.268
Node 10	2.103	-1.695	-1.033	-1.095	-1.500	1.225	3.150	-1.314	2.997
Node 11	-0.758	-1.932	0.455	1.141	-0.592	1.047	-0.798	-0.910	-2.493
Node 12	-0.512	-0.363	-0.837	-1.628	-1.254	-6.467	2.489	-2.325	6.244
Node 13	0.633	-1.654	0.168	-0.281	-0.665	-0.127	0.007	0.302	-1.609
Node 14	-1.987	1.317	0.396	-0.429	-0.668	-0.559	1.918	-0.757	2.596
Node 15	0.396	-1.653	0.188	0.601	-0.132	0.365	-6.245	4.265	-2.827
Node 16	-0.058	0.016	-0.031	0.856	0.036	0.440	-0.361	1.834	-1.407
Node 17	-0.384	-0.519	0.831	0.029	0.718	0.303	0.651	0.351	-1.265
Node 18	0.720	-0.717	-0.291	-0.613	0.301	0.600	5.014	-3.374	-4.533
Node 19	-0.142	-0.376	0.251	-0.346	0.503	0.482	0.295	0.592	-0.934
Node 20	0.102	-0.055	4.393	-7.944	-0.435	-0.164	-0.255	-5.112	5.368
Node 21	-0.266	-1.096	0.378	0.245	-1.350	-0.916	6.123	-2.640	-2.226
Node 22	0.103	0.396	-0.152	-0.193	-0.791	0.039	-0.602	-0.945	1.194
Node 23	-0.380	0.779	-0.203	2.070	-0.419	0.067	0.899	0.113	1.375
Node 24	0.720	0.262	0.621	0.039	-0.012	1.662	0.209	-0.564	2.934
Node 25	1.735	1.808	-0.899	-0.506	-1.345	1.951	-0.914	-1.099	-2.063

Supplementary Table 4

Additional neural network parameters as described in section 2.1.2.

α_1	0.141
α_2	0.131
α_3	0.054
α_4	0.081
b_O	-2.469
