# Supplementary material for: Gene prediction in metagenomic fragments: A large scale machine learning approach
Source: BMC Bioinformatics. 2008 Apr 28;9:217. doi: 10.1186/1471-2105-9-217 (PMC2409338; doi:10.1186/1471-2105-9-217)

Additional file 2 — Supplementary figures

Supplementary Figure 1: Discriminant validation

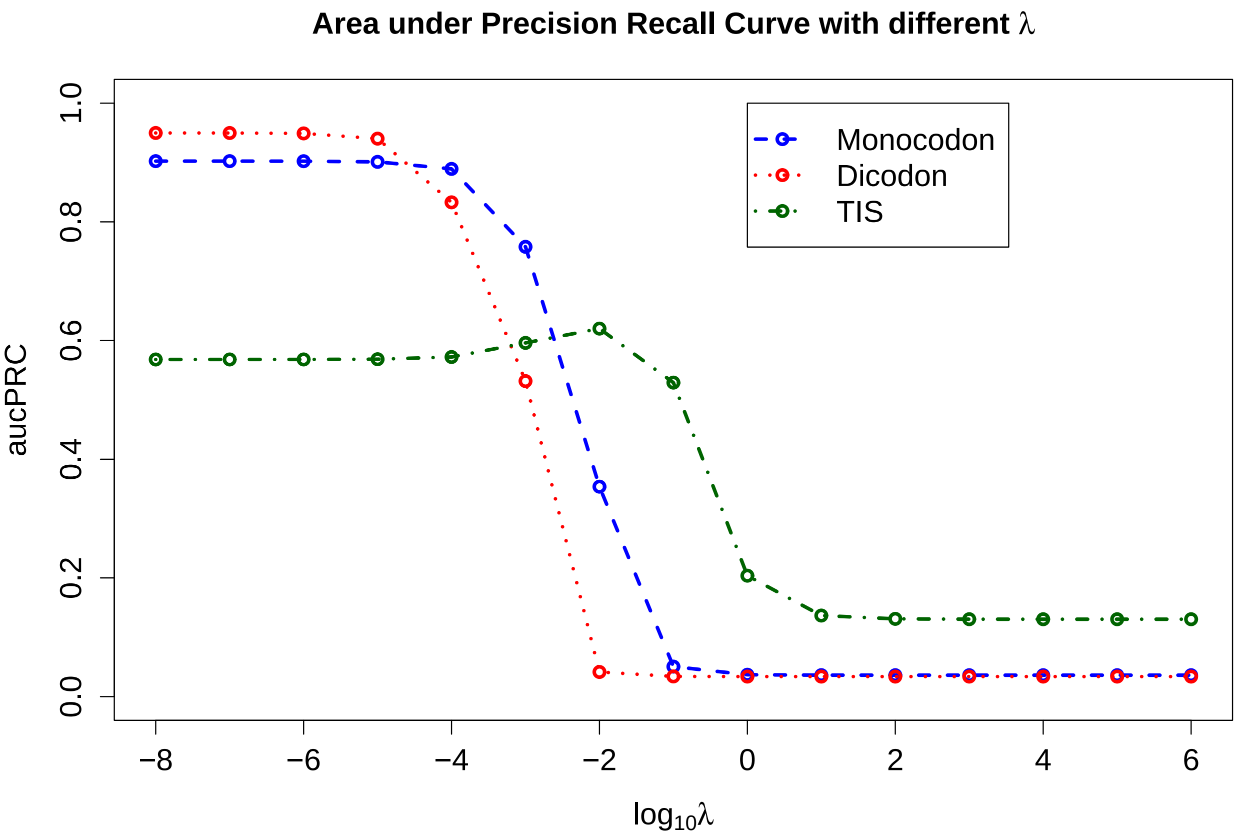

Supplementary Figure 2: Neural network performance

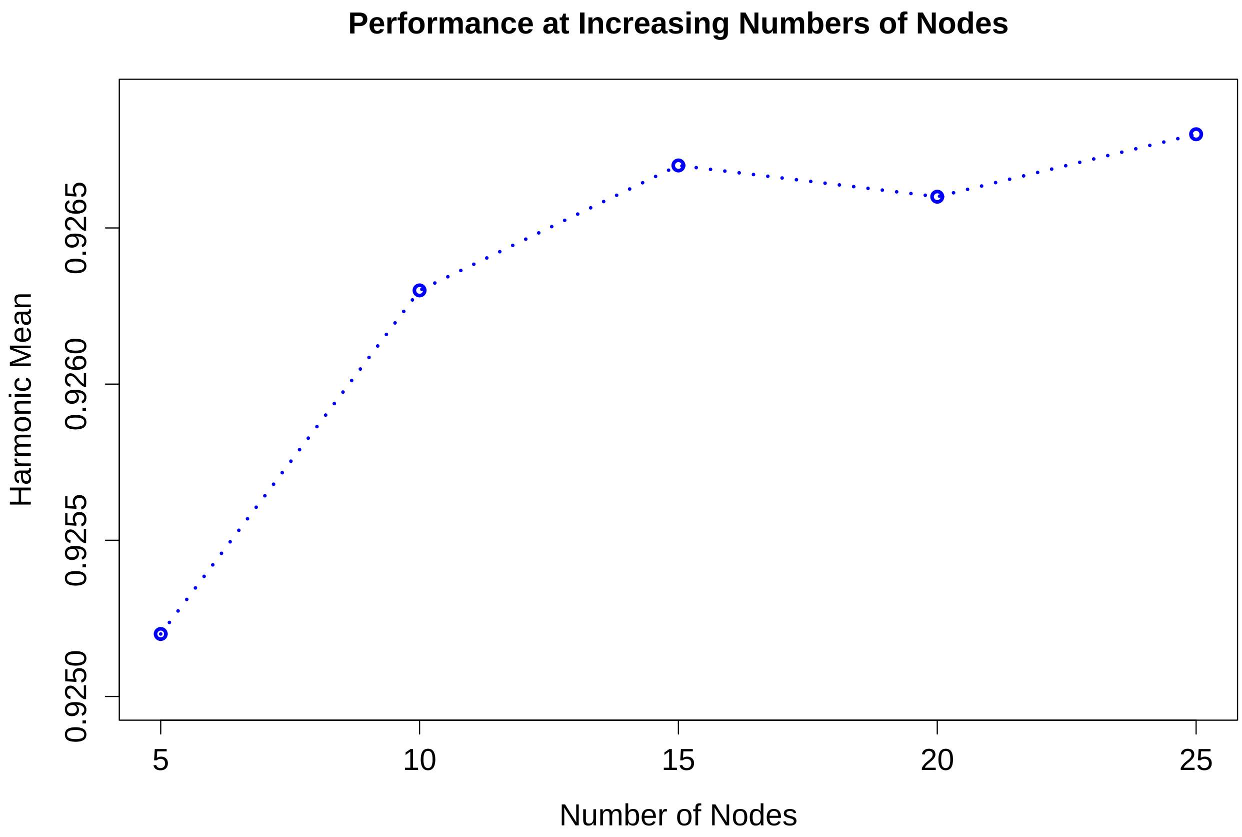

Supplementary Figure 3: Mean and standard deviation of the percentage of complete genes within all annotated genes per fragment averaged over all test-species fragments (with a 5-fold genome coverage, according to GenBank annotation).

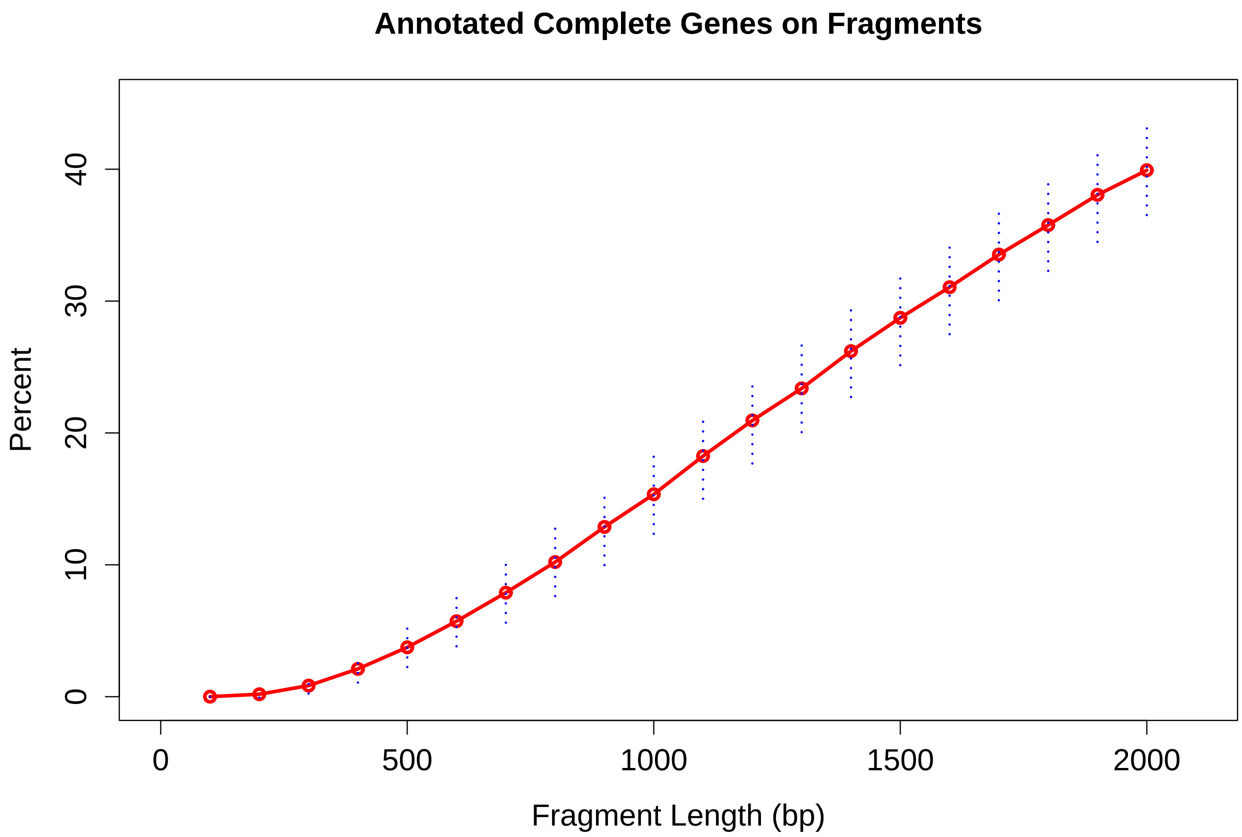

Supplementary Figure 4: Gene prediction performance on fragments ranging from 5000 to 60000 bp length (sampled to a 5-fold genomes coverage).

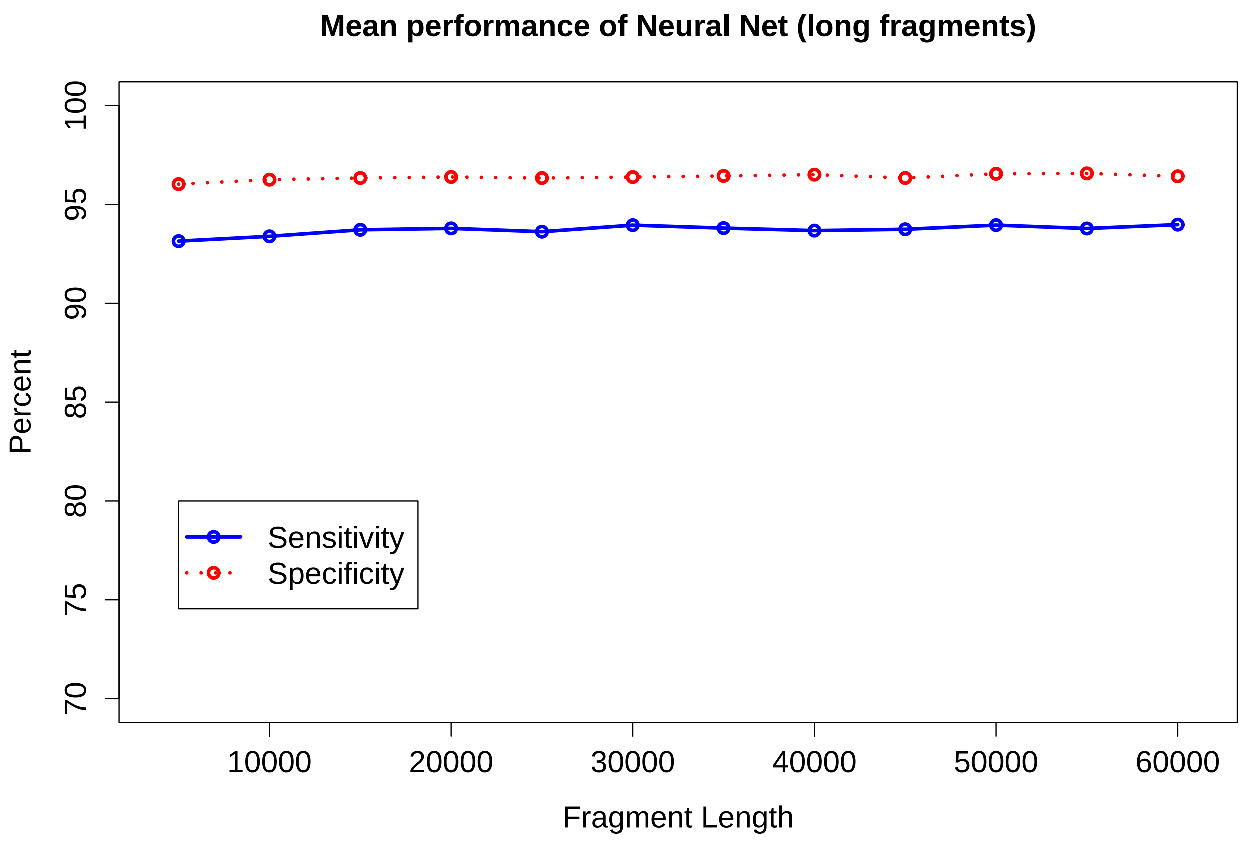

Supplement: Additional file 2 — Supplementary figures. The figures show the area under precision recall curve for discriminant validation using different λ values (1), the neural network performance with increasing numbers of nodes (2), the percentage of complete genes within all annotated genes per fragment for different fragment lengths (3), and gene prediction performance on fragments ranging from 5000 to 60000 bp (4). [file 1471-2105-9-217-S2.pdf]
